# The Role of Statistical Power: A Study of Relationship Between Emotional and Conduct Problems, Sociodemographic Factors, and Smoking Behaviours in Large and Small Samples of Latvian Adolescents

**DOI:** 10.3390/medicina61040687

**Published:** 2025-04-09

**Authors:** Viola Daniela Kiselova, Kristine Ozolina, Maksims Zolovs, Evija Nagle, Ieva Reine

**Affiliations:** 1 Institute of Public Health, Riga Stradins University, Kronvalda Boulevard 9, LV-1010 Riga, Latvia; kristine.ozolina@rsu.lv; 2 Statistics Unit, Riga Stradins University, LV-1007 Riga, Latvia; maksims.zolovs@rsu.lv (M.Z.); evija.nagle@rsu.lv (E.N.); ieva.reine@rsu.lv (I.R.); 3 Institute of Life Sciences and Technology, Daugavpils University, LV-5401 Daugavpils, Latvia; 4 Department of Public Health and Caring Sciences, Uppsala University, BMC, Husargatan 3, Box 564, 751 22 Uppsala, Sweden

**Keywords:** e-cigarette, conventional cigarette, smoking behaviours, adolescents, emotional and conduct problems, sociodemographic factors, sample size, statistical power, effect size

## Abstract

*Background and Objectives*: Adolescent smoking is influenced by sociodemographic and psychological factors, including emotional and conduct problems. Understanding how sample size impacts the interpretation of these associations is critical for improving study design and public health interventions. This study examines the relationships between smoking behaviours, sociodemographic factors, and emotional and conduct problems, focusing on how sample size affects statistical significance and effect size interpretation. *Materials and Methods*: Data from the Latvian Health Behaviour in School-aged Children study was analysed. Chi-square tests and logistic regression were conducted to evaluate associations between smoking behaviours, sociodemographic factors, and emotional and conduct problems. Analyses were performed on both a large general sample and ten smaller generated subsamples to compare the impact of sample size on statistical outcomes. *Results*: Age and conduct problems emerged as the most consistent predictors of adolescent smoking behaviours across large and small samples, while other predictors, such as family affluence and family structure, showed weaker and less consistent associations. A large sample produced significant results even for weak predictors. *Conclusions*: This study highlights the importance of integrating effect size interpretation with statistical significance, particularly in large datasets, to avoid overstating findings. By leveraging real-world data, it provides practical recommendations for improving study design and interpretation in behavioural, medical, and public health research, contributing to more effective interventions targeting adolescent smoking.

## 1. Introduction

The relationships between sociodemographic factors, psychological factors, including emotional and conduct problems, and smoking behaviours have been widely explored, particularly among adolescents. Adolescence is a critical period when individuals are more likely to engage in risky behaviours, one of which is smoking [1]. Various sociodemographic factors, such as socioeconomic status, family structure, and educational attainment, significantly influence smoking initiation and patterns [2,3,4]. Psychological conditions, including emotional and conduct problems, as well as anxiety, depression, and behavioural disorders, have also been identified as important predictors of smoking [5,6]. These associations are often examined at the national level using cross-sectional or longitudinal studies with representative samples. Such studies provide valuable insights into population-level trends and the distribution of risk factors, making them a cornerstone of adolescent health research [6,7,8].

However, the methodological approaches employed in such studies, particularly sample size, play an important role in shaping the observed relationships. Research has repeatedly demonstrated that larger sample sizes can lead to statistically significant results even for small effects, while smaller samples may fail to detect meaningful associations due to insufficient statistical power [9,10,11,12,13]. These findings emphasise the need to consider the relationship between sample size, *p*-values, and effect sizes when interpreting research outcomes. As Goel et al. (2024) discuss, the *p*-value’s sensitivity to sample size can result in exaggerated practical relevance in large samples or missed findings in smaller ones [13]. Although this problem has been recognised in the statistical literature, this study offers a novel contribution and underscores its practical significance for public health research. To reinforce this point, previous studies have highlighted the trade-off between statistical significance and practical relevance, demonstrating that effect size provides an important context for interpreting *p*-values. For example, Sullivan and Feinn (2012) argue that a statistically significant result may not be clinically or practically meaningful, highlighting the need to report and interpret effect sizes in conjunction with *p*-values [14]. Similarly, Cohen (1988) emphasised the importance of considering effect sizes in behavioural science [15], while Gigerenzer et al. (2004) criticised the over-reliance on *p*-values without considering effect sizes [16]. By incorporating these perspectives, this paper seeks to enhance the theoretical foundation of the discussion and contribute to a more nuanced interpretation of statistical results.

Appropriate sample sizes improve effect size precision and reproducibility [17,18,19], while inadequate sizes compromise validity [20]. Recent advancements have highlighted the need for careful sample size planning, particularly in complex analyses like mediation models [21]. Simulation-based methods and power analysis tools have been developed to aid researchers in determining the optimal sample size required to detect the effects with sufficient power. These tools are instrumental in mitigating the risks associated with underpowered or overpowered studies and enhancing the robustness of research findings [22].

Combining public health insights with a detailed exploration of statistical considerations would provide a better understanding of how sample size impacts the reliability and validity of statistical results. Such findings would not only offer valuable insights for researchers but also provide actionable recommendations to public health and medical specialists, helping to optimise the interpretation of statistical findings for more accurate and impactful decision-making for adolescent smoking prevention.

This study aims to investigate the association between sociodemographic and behavioural factors and adolescent smoking habits (including tobacco use, e-cigarette use, and dual use) and to evaluate how sample size influences statistical significance, effect size, interpretation, as well as the practical meaning of these research findings.

## 2. Materials and Methods

### 2.1. Study Design and Sampling Procedures

The data were obtained from the Health Behaviour in School-aged Children (HBSC) study, conducted in Latvia during the 2017/2018 academic year [23]. The study follows the international research protocol designed by the HBSC scientific working group [24]. It employs a cross-sectional study design and targets students from general education program schools aged 11, 13, and 15 years, corresponding to grades 5, 7, and 9 in the Latvian educational system.

The instrument used in the study is a validated standardised questionnaire covering various aspects of health.

A multi-stage cluster sampling method was used for the selection of schools and classes. Schools were randomly selected from the Latvian Ministry of Education and Science’s registry using probability proportional to size (PPS) sampling. Within each selected school, one class from grades 5, 7, and 9 was randomly chosen. All students from these classes who were present at school on the day of data collection and whose parents had not submitted a written refusal to participate were included in the study.

**Figure 1:**
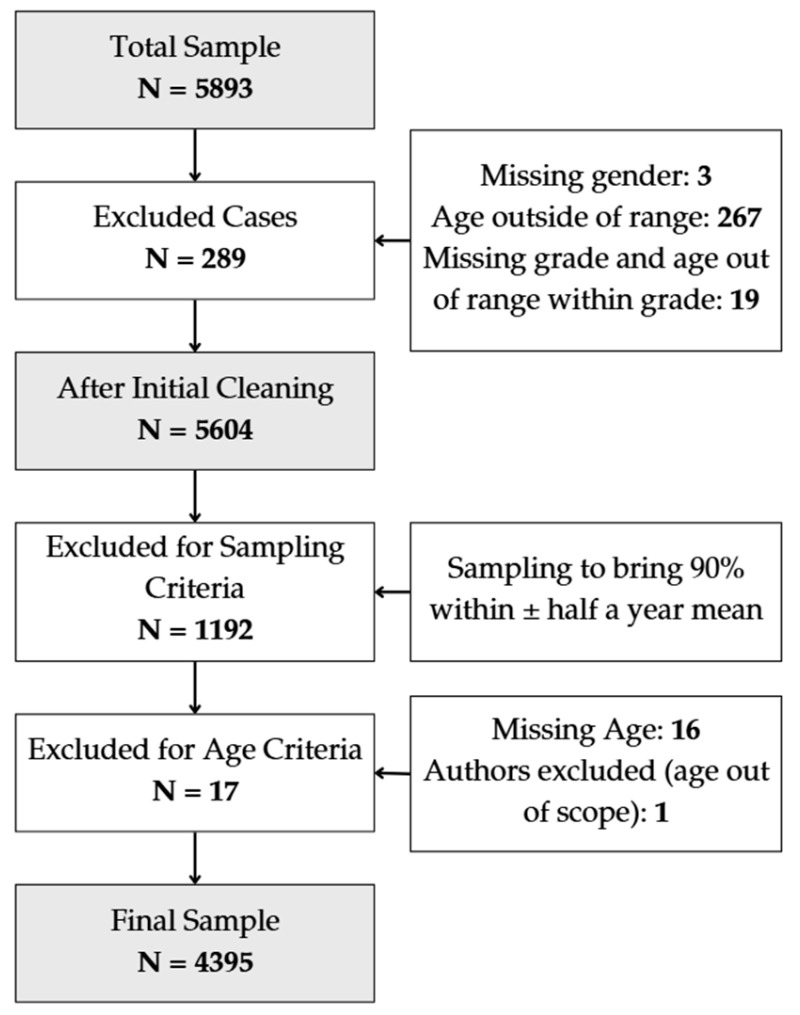
Flowchart of study participant inclusion/exclusion.

However, schools that follow special needs educational programs and schools, where the primary education language was neither Latvian nor Russian were excluded from the study. Also, respondents who left more than 25% of the questionnaire unanswered were excluded from the dataset through the initial data cleaning procedure. The final number of respondents in the dataset was 4395 students, with a response rate of 74% (Figure 1).

### 2.2. Measures

The key measures included in the analysis were sociodemographic factors, smoking behaviours, and emotional and conduct problems.

Sociodemographic factors included gender (boys and girls), age (categorized as 11, 13, and 15 years), family structure (living with both parents, one parent, or none), and level of financial security recoded into low, medium, and high-level categories based on responses to items from the Family Affluence Scale [25,26].

Smoking behaviours were assessed through questions about current cigarette and e-cigarette use, defined as having smoked or vaped at least one day in the past 30 days (current smokers). The dual smoking variable was created, capturing adolescents who used both cigarettes and e-cigarettes within the same period (at least one day in the past 30 days).

Emotional and conduct difficulties were assessed by using the Strengths and Difficulties Questionnaire (SDQ) [27]. Respondents rated 25 statements of the SDQ on a 3-point scale: “Not true”, “Somewhat true”, and “Certainly true”. Responses were divided into five subscales: emotional problems, conduct problems, hyperactivity, peer problems, and prosocial behaviour. For this study, the focus was on the emotional and conduct problems subscales. Based on previous research results in the Latvian population, the 90th percentile was used as the threshold to identify emotional and conduct difficulties [28]. Consequently, emotional problems were identified for respondents scoring 6 or higher on the emotional subscale, while conduct problems were identified for those adolescents who scored 7 or higher on the conduct problem subscale.

### 2.3. Statistical Analysis

Statistical analyses were conducted using descriptive and inferential methods. Frequency tables and cross-tabulations were used for initial data exploration, and Chi-square tests were applied to assess the relationships between categorical variables. The analysis was carried out using IBM SPSS version 27 and Jamovi version 2.4.11.

#### 2.3.1. General Sample Analysis

The general sample (n = 4395) served as the baseline for analysis. Descriptive statistics were calculated for all variables. Although age was recorded in three categories (11, 13, and 15 years), it was treated as a continuous variable in the analyses due to its log-linear relationship with the outcomes of interest, as the log-linearity assumption checks were performed. This treatment allowed for more precise modelling of age effects in regression analyses. Logistic regression was performed using a stepwise selection method, testing model performance based on the Akaike Information Criterion (AIC).

#### 2.3.2. Randomly Generated Subsamples

To examine the influence of the sample size, 10 smaller subsamples (n = 400) were randomly drawn from the general sample using the sample() function in Jamovi, with random selection applied. A fixed seed was not used, ensuring natural variation across samples.

The subsample size of n = 400 was selected based on a logistic regression requirement. Assuming an odds ratio of 2.0, a 10% event rate, a sample of ~350–400 was needed [29]. Additionally, n = 400 was selected based on descriptive research design where at 95% power (1 − β = 0.95) and a significance level of α = 0.05 a minimum sample size of approximately 400 participants would be required.

Each subsample underwent the same analytical procedures as the general sample:

Chi-square analyses were conducted, and effect sizes were calculated using Cramer’s V coefficient.

For each subsample, the average percentage for each category (e.g., smoking prevalence by gender) was calculated along with the maximum and minimum *p*-values, effect sizes, and the number of subsamples with *p*-values greater than 0.05.

Logistic regression models were fitted to assess the relationship between variables in the subsamples. To ensure comparability with the general sample, the same variables used in the general sample’s logistic regression model were applied to the subsamples, regardless of AIC differences across subsamples. This approach ensured that the results were directly comparable, and differences were attributed solely to sample size rather than variations in model specification.

#### 2.3.3. Consolidated Subsample Results

For consolidated results across the ten subsamples, average ORs, 95% CI lower and upper bounds (excluding cases with infinite values), average percentages for each category, average and range (minimum and maximum) of *p*-values, effect sizes (Cramer’s V) and their range (minimum and maximum), count of subsamples where *p* > 0.05 were calculated using Excel (Version 2411).

#### 2.3.4. Effect Size Measures and Comparison Across Samples

To assess the strength of associations and their practical significance, effect size measures were employed alongside statistical significance tests, recognising their dependency on sample size. Cramer’s V was calculated for Chi-square analyses to quantify the strength of associations between categorical variables, with thresholds interpreted: coefficient below 0.30 was considered as weak association, 0.30 to 0.49—moderate association, and 0.50 and above—a strong association based on Cohen (1988) [15]. Binary logistic regression was used to examine the associations between smoking behaviours and predictor variables. Given the cross-sectional nature of the study, odds ratios (ORs) were selected as the measure of association, as they are standard in such designs where temporal order cannot be established. Logistic regression is also appropriate for binary outcomes and allows for straightforward interpretation of results in the context of public health and behavioural research. ORs close to 1 indicate weak effects and larger deviations suggest stronger associations.

McFadden’s R^2^ was used to evaluate the overall fit of logistic regression models, with values typically ranging from 0 to 1; higher values indicate better fit, though in logistic regression, values above 0.2 are often considered indicative of good to excellent fit [30,31].

Although pseudo R^2^ values, such as McFadden’s R^2^, are generally low in logistic regression and should not be interpreted in the same way as traditional R^2^ values in linear models, they can still serve as a useful tool for comparing the relative fit of similar models [31]. In this study, McFadden’s R^2^ was used to provide a general indication of explanatory strength and to compare model performance across different sample sizes.

For logistic regression analysis—girls were used as the reference category, for emotional and conduct problems—those who had no such problems were identified, for age—11-year-olds, for family structure—both parents, and for family affluence level—low family affluence.

To address the sample size issue, we compared results across a large general sample (n = 4395) and ten smaller subsamples (n = 400 each), reporting both *p*-values and effect sizes to ensure a balanced interpretation. No formal significance corrections were applied, as the study aimed to explore sample size effects rather than control for multiple testing across independent hypotheses. Instead, sensitivity to sample size was examined by consolidating subsample results (e.g., averaging ORs and effect sizes, reporting *p*-value ranges) to highlight variability and stability of findings.

Missing data were excluded from all analyses. Results were interpreted with a 95% confidence level to ensure statistical robustness. Confidence intervals for categorical outcomes were calculated using the Wilson method to account for sample variability.

## 3. Results

### 3.1. Characteristics of the General Sample

The representative sample of the study (Table 1) included 4395 Latvian adolescents aged 11, 13 and 15 years with a nearly equal distribution of boys and girls.

Based on SDQ results, in this study sample, 11.2% of adolescents reported experiencing emotional problems. A similar prevalence was obtained in the conduct problem scale—10.9%.

When examining smoking behaviours, 9.6% of participants reported using e-cigarettes at least one day in the last 30 days, while 10.2% reported currently smoking conventional cigarettes. Additionally, 4.8% smoked both e-cigarettes and conventional cigarettes in the past 30 days.

### 3.2. Association Analysis

The association between gender and smoking behaviours was examined in the general study sample and a consolidated sample derived from ten randomly generated smaller samples of the general sample. Significant results were observed for current e-cigarette use and dual smoking. In the general sample, boys had a significantly higher prevalence of current e-cigarette use (12.5%) compared to girls (6.8%, *p* < 0.001, Cramer’s V = 0.097). In the consolidated sample, the association remained significant in eight out of ten smaller samples (*p* = 0.025 overall), with effect sizes ranging from 0.004 to 0.068 (Cramer’s V = 0.122). Boys were significantly more likely to report dual smoking compared to girls in the general sample (5.8% vs. 3.9%, *p* = 0.004, Cramer’s V = 0.044). In the consolidated sample, the association was significant in one out of ten smaller samples (*p* = 0.299 overall), with effect sizes ranging from 0.012 to 0.812 (Cramer’s V = 0.066). Current cigarette smoking showed no significant association with gender in either sample (Table 2).

Family affluence showed no significant association with current e-cigarette use (*p* = 0.574, Cramer’s V = 0.016) or dual smoking (*p* = 0.139, Cramer’s V = 0.031) in the general sample, with all ten consolidated subsamples being insignificant for dual use and eight for e-cigarette use. A weak but significant association was found for current cigarette smoking in the general sample (*p* = 0.005, Cramer’s V = 0.050), though nine generated subsamples showed insignificant association (*p* = 0.503, Cramer’s V = 0.064) (Table 3).

Family structure was significantly associated with current e-cigarette use (*p* < 0.001, Cramer’s V = 0.099), current cigarette smoking (*p* < 0.001, Cramer’s V = 0.110), and dual smoking (*p* < 0.001, Cramer’s V = 0.088) in the general sample, with all effect sizes indicating weak associations (Table 4). Smoking prevalence was highest among individuals from single-parent households. In the consolidated sample, the results were less consistent, with seven subsamples showing insignificant associations for e-cigarette and cigarette smoking, and eight for dual smoking.

### 3.3. Logistic Regression Results

Logistic regression models were performed to assess predictors of current e-cigarette use, cigarette smoking, and dual smoking behaviours in both the general study sample and the consolidated sample from the study group. The results, including deviance, McFadden’s R^2^, AIC, and *p*-values, are summarised in Table 5.

All models were statistically significant (*p* < 0.05) for both general and consolidated samples, indicating that the predictors contributed meaningfully to the outcomes.

The consolidated sample consistently showed better fit, as indicated by higher McFadden’s R^2^. For current e-cigarette use, R^2^_McF_ increased from 0.125 (general sample) to 0.164 (consolidated sample). For current cigarette smoking, it increased from 0.140 to 0.185. For current dual smoking, it increased from 0.106 to 0.189.

Deviance and AIC values are reported for transparency, but they are not directly compared between samples due to their dependency on sample size, which affects their calculation.

The binomial logistic regression analysis identified key predictors of adolescent smoking behaviours, with notable variability between the general and consolidated samples (Table 6).

Boys, compared to girls, had higher odds of current e-cigarette use (OR = 2.115, 95% CI: 1.679–2.660) and dual smoking (OR = 1.676, 95% CI: 1.239–2.270) in the general sample. Age was consistently significantly associated with increased odds of smoking, for example for e-cigarette use OR = 1.677 in the general sample and OR = 1.725 in the consolidated sample.

Adolescents from single-parent households had higher odds of current e-cigarette use (OR = 1.801, 95% CI: 1.436–2.260) and cigarette smoking (OR = 1.833, 95% CI: 1.474–2.280) in the general sample. These associations weakened in the consolidated sample, with wide confidence intervals and non-significant results.

Conduct problems strongly predicted smoking behaviours, with OR = 2.773 for e-cigarette use (95% CI: 2.087–3.680) and OR = 2.240 for dual smoking (95% CI: 1.545–3.250) in the general sample. These associations were less consistent in the consolidated sample, showing insignificant association (*p* > 0.05).

## 4. Discussion

### 4.1. Associations Between Sociodemographic Factors, Emotional and Conduct Problems, and Smoking Behaviours

This study examined the relationships between smoking behaviours and various sociodemographic factors, as well as emotional and conduct problems among adolescents.

In the general sample, gender and age were consistent predictors of smoking, aligning with prior studies [32,33,34]. Boys had higher odds of e-cigarette use (OR = 2.115, 95% CI: 1.679–2.660) and dual smoking (OR = 1.677, 95% CI: 1.239–2.270) than girls, though dual-use associations weakened in the consolidated sample, suggesting sensitivity to sample size. Age remained a strong predictor across samples (ORs = 1.7–2.1), highlighting the rising probability with age [33,34]. In the general sample, adolescents from single-parent households had higher odds of current e-cigarette use, cigarette smoking and dual smoking compared to those living with both parents. However, these associations became weaker and non-significant—with wider confidence intervals in the consolidated sample, suggesting that while family structure plays a role in adolescent smoking, its impact may have limited practical significance compared to more robust predictors like age and may be less consistent than previously thought. The loss of significance in smaller samples reveals the potential influence of other contextual factors, such as parental supervision, socioeconomic stability, and peer relationships [2,35,36,37], which were not measured in this study. Public health interventions should consider these broader social determinants when addressing smoking behaviour disparities in adolescents from different family backgrounds.

Among other predictors, conduct problems showed the strongest and most stable association with smoking behaviours for the general sample. However, in the consolidated sample, the associations weakened, with wider confidence intervals and *p*-values suggesting non-significant results. Although other studies link externalising problems to risk-taking behaviours, including smoking [6,38], in the consolidated sample we did not detect a significant association.

Emotional problems and family affluence showed weak and inconsistent associations with smoking, where odds ratios were close to value one and wide confidence intervals were observed. While emotional difficulties have been linked to self-medication theories of substance use [39], our findings do not provide strong support for the emotional problem relationship with smoking behaviours. Similarly, the lack of a clear association between family affluence and smoking suggests that economic resources alone may not be a direct protective or risk factor. Instead, other factors such as family support, peer pressure, social cohesion, and access to positive coping mechanisms may play a more critical role [1,35,36,37,40]. Future research should explore these psychosocial dimensions to better understand the pathways linking socioeconomic status and mental health to adolescent smoking behaviours.

From a public health perspective, the consistent role of age suggests that targeted interventions should address the fact that adolescence is a period of vulnerability to risky behaviours. Furthermore, these findings highlight the importance of designing age-specific interventions, given the developmental transitions during adolescence that contribute to smoking initiation.

### 4.2. The Effect of Sample Size on Statistical Estimates

In the initial phase of this study, exploratory data analysis, and logistic regression were conducted to examine the associations between smoking behaviours and sociodemographic and emotional and conduct problems, as well as to analyse the mediating role of emotional and conduct problems in the relationship between sociodemographic factors and smoking behaviours. Although the analyses revealed statistically significant results, the effect sizes were modest. These findings prompted a critical evaluation of the study’s design, particularly concerning sample size adequacy. Therefore, the role of sample size in shaping statistical results was evident in this study.

The choice of sample size significantly influences the conclusions drawn from this study. While the general sample provided stronger statistical power to detect associations, the smaller subsamples revealed inconsistencies, affecting the reliability and interpretation of findings. This pattern aligns with theoretical insights into the relationship between sample size, statistical power, and *p*-values [41].

In the general sample, numerous smoking predictors—gender, age, family structure, and conduct problems—were statistically significant, largely due to the increased power provided by a large sample size. However, despite the statistical significance, all effect sizes were weak, suggesting that while associations exist, their real-world impact may be minimal [42]. The large sample likely inflated the detection of weak associations, meaning that statistically significant results should be interpreted cautiously in terms of practical relevance [43].

When using smaller subsamples, several associations weakened or became non-significant. For example, the association between family structure and e-cigarettes, which was significant in the general sample, was no longer significant in the consolidated sample (nine out of ten generated subsamples showed insignificant association). Similarly, conduct problems remained a strong predictor in the general sample, but lost significance in the smaller subsamples, likely due to increased variability and wider confidence intervals [44].

These inconsistencies suggest that some associations observed in the general sample may not be stable or generalisable across different datasets. The loss of statistical significance in smaller samples indicates that these relationships might be dependent on sample composition and size, rather than representing strong, underlying effects.

A key consideration in statistical analysis is the influence of sample size on effect size stability and significance testing. In this study, we focused on analysing subsamples of n = 400 to assess the robustness of statistical associations in smaller datasets. However, smaller sample sizes (e.g., n = 100) may result in increased variability and reduced statistical power, leading to a higher likelihood of Type II errors [44]. Conversely, larger samples (e.g., n = 1000) may yield statistically significant results for effects that are practically negligible, increasing the risk of Type I errors [13].

This study reinforces the need for researchers to report and interpret effect sizes alongside *p*-values, particularly when working with large datasets. In this context, smaller samples with adequate power may yield more realistic estimates of effect sizes, reducing the risk of overemphasising minor associations, as supported by prior studies [45].

### 4.3. Strengths and Limitations of the Study

One of the primary strengths of this study is the availability of a large, nationally representative dataset, which enabled robust comparisons between large and small samples. The use of real-world data enhances the relevance and applicability of the findings to public health research. Additionally, the focus on effect sizes alongside *p*-values provides a more nuanced understanding of the associations, addressing a common limitation in studies using large datasets.

Despite its strengths, this study has several limitations. The study’s cross-sectional design limits causal interpretations of the observed associations. The use of consolidated subsamples revealed inconsistencies, suggesting that the large general sample may overestimate the significance of weak predictors due to increased statistical power. Self-reported data on substance use and other variables may be subject to recall bias or social desirability bias, potentially affecting the reliability of the findings. While the evidence supports the influence of sociodemographic factors and mental health issues on adolescent smoking, it is essential to consider that not all adolescents with similar backgrounds or emotional problems will engage in smoking. Individual resilience and protective factors, such as supportive family environments and positive peer relationships, which were not measured in this study, can potentially mitigate these risks [2,35,36,37].

### 4.4. Recommendations and Future Directions

The findings of this study underline the importance of integrating effect size interpretation with statistical significance, particularly when working with large datasets. While large samples offer the advantage of detecting subtle associations, the risk of overinterpreting weak effect sizes must be addressed. Researchers should prioritize reporting both statistical significance and effect sizes to provide a balanced understanding of results and avoid exaggerating the practical implications of minor associations.

The current results align with a growing body of literature cautioning against the overinterpretation of statistically significant results in large samples where effect sizes remain small (e.g., Peeters, 2016 [42]; Gómez-de-Mariscal et al., 2021 [41]; Lantz, 2013 [43]). In particular, Goel et al. (2024) [13] highlight the *p*-value’s sensitivity to sample size, underlining the importance of reporting and interpreting effect sizes to avoid misleading conclusions. As suggested by recent methodological studies, incorporating simulation-based approaches or Bayesian frameworks can offer more nuanced interpretations that go beyond *p*-values [22,45]. While this study did not adopt formal Bayesian inference, it used consolidated subsample analysis as a pragmatic alternative to explore effect stability. This strategy revealed that several predictors lost significance in smaller samples despite large-sample significance—emphasising the importance of replication, effect size magnitude, and confidence intervals in evaluating practical relevance. Future research may benefit from applying Bayesian or simulation-based methodologies to further strengthen inference, particularly when studying behaviours with modest effect sizes and high contextual variability.

Another essential consideration for future research is the use of appropriate sample size calculations during the study design phase. Since many studies have limited resources (e.g., funding, time, and personnel), it is crucial to calculate the optimal sample size before data collection to avoid unnecessary expenditures. Power analyses should be conducted to determine the optimal sample size required to detect meaningful associations with adequate statistical power. This approach helps avoid underpowered studies, which are prone to Type II errors, and overpowered studies, which may produce significant results for trivial effects [9]. By aligning sample size with research objectives, studies can produce more robust and actionable results.

Given the limitations of traditional statistical approaches in handling large datasets with weak effect sizes, incorporating machine learning techniques presents an opportunity for future research. Machine learning algorithms, such as random forests, support vector machines, and neural networks, can capture complex, non-linear relationships that might be overlooked by conventional models [46]. These methods may also provide insights into interactions between sociodemographic and psychological factors, offering a deeper understanding of predictors of adolescent smoking behaviours.

Future studies should also consider the inclusion of protective factors to provide a more comprehensive understanding of the factors influencing smoking behaviours [35,36,37]. Stratified analyses and subgroup-specific modelling could offer valuable insights into how these relationships differ across contexts and populations.

Moreover, longitudinal studies are essential to establish causal pathways between sociodemographic factors, emotional and conduct problems, and smoking behaviours. Exploring dynamics through such designs would strengthen the evidence base for targeted public health interventions.

Finally, as the field advances, researchers and practitioners should focus on translating findings into actionable interventions. For example, interventions can be tailored to specific age groups by using insights gained from large datasets and advanced analytical methods. By leveraging both traditional statistical techniques and emerging machine learning approaches, future research can optimise the use of large datasets to enhance the reproducibility and applicability of findings, contributing to more effective public health strategies to prevent adolescent smoking.

## 5. Conclusions

This study illustrates the influence of sample size on the interpretation of associations between sociodemographic factors, emotional and conduct problems, and smoking behaviours. Age and conduct problems emerged as key predictors, highlighting their importance for public health interventions.

Furthermore, this study demonstrates how sample size critically influences the estimation and interpretation of statistical associations in behavioural research. While large samples enhance statistical power and increase the likelihood of detecting significant effects, they may also amplify the detection of associations that are statistically significant but practically negligible. By comparing a general population sample with multiple smaller subsamples, we illustrated how the strength and stability of predictors—such as those related to smoking behaviour—can vary depending on sample size.

To provide a more nuanced interpretation, we employed effect size measures such as odds ratios and Cramer’s V alongside traditional significance testing. This approach enabled us to distinguish between statistical and practical significance and highlighted the importance of presenting confidence intervals in addition to *p*-values.

These findings emphasize the need for researchers to interpret statistically significant results with caution, particularly in large datasets, and to consistently report and discuss effect sizes. From a health policy and public health perspective, our results support a shift toward effect size-driven interpretation, particularly in contexts where practical implications outweigh marginal statistical gains. Ultimately, this study contributes to the growing literature advocating for more transparent, balanced, and context-aware reporting in quantitative research.

## Figures and Tables

**Table 1:** General sample characteristics.

Variable	Category	Count, n	Proportion, %	95% CI ^1^
Gender	Boys	2179	49.6	48.1–51.1
	Girls	2216	50.4	48.9–51.9
Age	11 years	1534	34.9	33.5–36.3
	13 years	1519	34.6	33.2–36.0
	15 years	1342	30.5	29.2–31.9
Family structure	Both parents	2739	62.3	60.9–63.7
	One parent	1432	32.6	31.2–34.0
	None	224	5.1	4.5–5.8
Family affluence	High	2357	54.8	53.3–56.3
	Medium	1760	40.9	39.5–42.4
	Low	181	4.2	3.7–4.9
Emotional problems	Identified problems	484	11.2	10.3–12.2
	No identified problems	3821	88.8	87.8–89.7
Conduct problems	Identified problems	471	10.9	10.0–11.9
	No identified problems	3839	89.1	88.1–90.0
Current e-cigarette use	Used at least once in the last 30 days	403	9.6	8.7–10.5
	Did not use in the last 30 days	3798	90.4	89.5–91.3
Current cigarette smoking	Smoked at least once in the last 30 days	439	10.2	9.3–11.1
	Did not smoke in the last 30 days	3872	89.8	88.9–90.7
Dual smoking	Smoked at least once in the last 30 days	209	4.8	4.2–5.5
	Did not smoke in the last 30 days	4124	95.2	94.5–95.8

^1^ Confidence interval.

**Table 2:** The difference in smoking behaviour between gender groups.

Dependent Variable	Gender		General Study Sample		Consolidated Sample from the Study Group			
	Boys	Girls	*p* Value	Effect Size (Cramer’s V)	*p* Value	*p* Value Range	Effect Size (Cramer’s V)	Effect Size Range
Current e-cigarette use (n, %)			<0.001	0.097	0.025	0.004–0.068	0.122	0.094–0.149
Used in the past 30 days	258, 12.5%	145, 6.8%						
Did not use in the past 30 days	1809, 87.5%	1989, 93.2%						
Current cigarette smoking (n, %)			0.725	0.005	0.622	0.308–0.988	0.122	0.001–0.052
Smoked in the past 30 days	213, 10.0%	226, 10.3%						
Did not smoke in the past 30 days	1913, 90.0%	1959, 89.7%						
Current dual smoking (n, %)			0.004	0.044	0.299	0.012–0.812	0.066	0.012–0.128
Smoked in the past 30 days	124, 5.8%	85, 3.9%						
Did not smoke in the past 30 days	2023, 94.2%	2101, 96.1%						

**Table 3:** Difference in smoking behaviour between family affluence level groups.

Dependent Variable	Family Affluence			General Study Sample		Consolidated Sample from the Study Group			
	High	Medium	Low	*p* Value	Effect Size (Cramer’s V)	*p* Value	*p* Value Range	Effect Size (Cramer’s V)	Effect Size Range
Current e-cigarette use (n, %)				0.574	0.016	0.377	0.003–0.940	0.016	0.018–0.202
Used in the past 30 days	210, 9.3%	167, 9.9%	20, 11.6%						
Did not use in the past 30 days	2041, 90.7%	1520, 90.1%	153, 88.4%						
Current smoking (n, %)				0.005	0.050	0.503	0.024–1.000	0.064	0.027–0.149
Smoked in the past 30 days	208, 9.0%	199, 11.5%	26, 14.7%						
Did not smoke in the past 30 days	2101, 91.0%	1531, 88.5%	151, 85.3%						
Both (n, %)				0.139	0.031	0.516	0.088–0.896	0.063	0.024–0.110
Did in the past 30 days	106, 4.6%	85, 4.9%	14, 7.9%						
Did not in the past 30 days	2218, 95.4%	1652, 95.1%	164, 92.1%						

**Table 4:** Difference in smoking behaviour between family structure groups.

Dependent Variable	Family Structure			General Study Sample		Consolidated Sample from the Study Group			
	One Parent	Both	None	*p* Value	Effect Size (Cramer’s V)	*p* Value	*p* Value Range	Effect Size (Cramer’s V)	Effect Size Range
Current e-cigarette use (n, %)				<0.001	0.099	0.257	0.007–0.785	0.102	0.028–0.165
Used in the past 30 days	188, 13.8%	196, 7.5%	19, 9.0%						
Did not use in the past 30 days	1178, 86.2%	2428, 92.5%	192, 91.0%						
Current cigarette smoking (n, %)				<0.001	0.110	0.243	0.001–0.785	0.105	0.046–0.199
Smoked in the past 30 days	210, 15.0%	209, 7.8%	20, 9.1%						
Did not smoke in the past 30 days	1193, 85.0%	2479, 92.2%	200, 90.9%						
Current dual smoking (n, %)				<0.001	0.088	0.357	0.005–1.000	0.092	0.032–0.162
Smoked in the past 30 days	106, 7.5%	93, 3.4%	10, 4.5%						
Did not smoke in the past 30 days	1302, 92.5%	2610, 96.6%	212, 95.5%						

**Table 5:** Binomial logistic regression model fit coefficients.

Dependent Variable	Sample	Deviance	AIC	R^2^_McF_	*p* Value
Current e-cigarette use	General study sample	2258	2276	0.125	<0.001
	Consolidated sample from the study group	186	204	0.164	0.001
Current cigarette smoking	General study sample	2378	2396	0.140	<0.001
	Consolidated sample from the study group	196	219	0.185	0.001
Current dual smoking	General study sample	1453	1471	0.106	<0.001
	Consolidated sample from the study group	108	126	0.189	0.0244

**Table 6:** Binomial logistic regression analysis of sociodemographic factors and emotional and conduct problem association with current e-cigarette use, conventional cigarette, and dual smoking.

Dependent Variable	Parameters	General Study Sample			Consolidated Sample from the Study Group			
		Odds Ratio	95% CI ^1^	*p*-Value	Odds Ratio	95% CI	*p*-Value	*p*-Value Range ^3^
Current e-cigarette use	Gender							
	Boys–Girls	2.115	1.679–2.660	<0.001	2.765	1.191–6.435	0.035	<0.001–0.129
	Age	1.677	1.549–1.820	<0.001	1.725	1.300–2.290	0.001	<0.001–0.002
	Family affluence							
	High–low	1.083	0.639–1.840	0.768	3.180 × 10^6^	0.112–Inf ^2^	0.515	0.053–0.992
	Medium–low	0.921	0.542–1.570	0.763	2.81 × 10^6^	0.060–Inf ^2^	0.562	0.061–0.992
	Family structure							
	One parent–both parents	1.801	1.436–2.260	<0.001	1.785	0.795–4.027	0.388	<0.001–0.816
	No parents–both parents	0.879	0.517–1.490	0.634	0.573	0.107–Inf ^2^	0.705	0.258–0.992
	Emotional problems							
	Identified problems–no identified problems	1.245	0.891–1.740	0.199	1.116	0.326–4.001	0.326	0.084–0.684
	Conduct problems							
	Identified problems–no identified problems	2.773	2.087–3.680	<0.001	3.168	1.074–9.482	0.181	0.001–0.792
Current cigarette use	Gender							
	Boys–Girls	1.002	0.807–1.240	0.986	1.081	0.498–2.348	0.559	0.340–0.943
	Age	1.872	1.726–2.030	<0.001	2.137	1.557–2.937	<0.001	<0.001–<0.001
	Family affluence							
	High–low	0.785	0.488–1.260	0.319	4.110 × 10^5^	0.151–Inf ^2^	0.571	0.05–0.988
	Medium–low	0.800	0.497–1.290	0.358	4.440 × 10^5^	0.137–Inf ^2^	0.560	0.068–0.988
	Family structure							
	One parent–both parents	1.833	1.474–2.280	<0.001	1.696	0.782–3.688	0.388	0.01–0.976
	No parents–both parents	0.940	0.562–1.570	0.813	0.838	0.166–Inf ^2^	0.759	0.15–0.988
	Emotional problems							
	Identified problems–no identified problems	1.370	1.013–1.850	0.041	1.789	0.630–5.138	0.318	0.01–0.762
	Conduct problems							
	Identified problems–no identified problems	2.207	1.652–2.950	<0.001	2.486	0.859–7.261	0.194	0.01–0.660
Current dual smoking	Gender							
	Boys–Girls	1.677	1.239–2.270	<0.001	2.800	0.774–10.525	0.315	0.019–0.855
	Age	1.676	1.504–1.870	<0.001	1.947	1.232–3.105	0.030	<0.001–0.209
	Family affluence							
	High–low	0.757	0.413–1.390	0.369	3.900 × 10^6^	0.048–Inf ^2^	0.658	0.018–0.995
	Medium–low	0.651	0.353–1.200	0.169	2.287 × 10^6^	0.034–Inf ^2^	0.573	0.018–0.995
	Family structure							
	One parent–both parents	2.036	1.509–2.750	<0.001	2.432	0.710–8.498	0.432	0.005–0.964
	No parents–both parents	1.052	0.530–2.090	0.884	0.775	0.086–Inf ^2^	0.836	0.433–0.995
	Emotional problems							
	Identified problems–no identified problems	1.502	0.995–2.270	0.053	2.045	0.452–Inf ^2^	0.439	0.012–0.991
	Conduct problems							
	Identified problems–no identified problems	2.240	1.545–3.250	<0.001	3.391	0.859–14.177	0.418	0.006–0.989

^1^ Confidence interval. ^2^ The mean value could not be estimated as the 95% confidence interval for one or more generated samples included an upper limit approaching infinity. ^3^ Minimum and maximum *p*-values were calculated from the generated study samples.

## Data Availability

Restrictions apply to the availability of these data. Data were obtained from the Centre for Disease Prevention and Control of Latvia and are available from the authors with the permission of the Centre for Disease Prevention and Control of Latvia.
